# Retraction: Knockdown of TMPRSS11D inhibits the proliferation, migration and invasion of cervical cancer cells

**DOI:** 10.1039/d1ra90048j

**Published:** 2021-01-27

**Authors:** Laura Fisher

**Affiliations:** Royal Society of Chemistry Thomas Graham House, Science Park, Milton Road Cambridge CB4 0WF UK advances-rsc@rsc.org

## Abstract

Retraction of ‘Knockdown of TMPRSS11D inhibits the proliferation, migration and invasion of cervical cancer cells’ by Kun Yan *et al.*, *RSC Adv.*, 2019, **9**, 21591–21600, DOI: 10.1039/C9RA02482D.

The Royal Society of Chemistry hereby wholly retracts this *RSC Advances* article due to concerns with the reliability of the data. The images in the article, and the raw data provided by the authors, were screened by an image integrity expert. The western blot bands have a very regular formulaic shape that is unlikely to be genuine. In addition, while the original data provided by the authors matched the figures, the images in the raw data were of very low quality and do not look genuine. Therefore, the raw data cannot be used to verify the published data. Furthermore, the raw data provided by the authors was found to closely resemble raw data provided for a number of other articles, which is unexpected given that there are completely different author lists for these articles.

Given the significance of the concerns about the validity of both the data in the article and the raw data provided by the authors, the findings presented in this paper are not reliable.

The authors have been informed but have not responded to any correspondence regarding the retraction.

Signed: Laura Fisher, Executive Editor, *RSC Advances*

Date: 15^th^ January 2021

## Supplementary Material

